# Ancestry and adaptive evolution of anadromous, resident, and adfluvial rainbow trout (*Oncorhynchus mykiss*) in the San Francisco bay area: application of adaptive genomic variation to conservation in a highly impacted landscape

**DOI:** 10.1111/eva.12416

**Published:** 2016-10-27

**Authors:** Maeva Leitwein, John Carlos Garza, Devon E Pearse

**Affiliations:** ^1^ Technopôle Brest‐Iroiserue Dumont d'UrvilleInstitut Universitaire Européen de la Mer (IUEM)University of BrestPlouzanéFrance; ^2^ Institute of Marine SciencesUniversity of CaliforniaSanta CruzCAUSA; ^3^ Fisheries Ecology DivisionSouthwest Fisheries Science CenterNational Marine Fisheries ServiceSanta CruzCAUSA; ^4^ Present address: Institut des Sciences de l'Evolution de Montpellier (ISEM)UMR 5554Université de ´MontpellierMontpellierCedex 5France

**Keywords:** adaptive genomic variation, conservation, evolution, life history, steelhead

## Abstract

The streams draining of into San Francisco Bay, California, have been impacted by habitat alteration for over 150 years, and roads, dams, water diversions, and other impediments now block the paths of many aquatic migratory species. These changes can affect the genetic structure of fish populations, as well as driving adaptive evolution to novel environmental conditions. Here, we determine the evolutionary relationships of San Francisco Bay Area steelhead/rainbow trout (*Oncorhynchus mykiss*) populations and show that (i) they are more closely related to native coastal steelhead than to the California Central Valley lineage, with no evidence of introgression by domesticated hatchery rainbow trout, (ii) populations above and below barriers within watersheds are each other's closest relatives, and (iii) adaptive genomic variation associated with migratory life‐history traits in *O. mykiss* shows substantial evolutionary differences between fish above and below dams. These findings support continued habitat restoration and protection of San Francisco Bay Area *O. mykiss* populations and demonstrate that ecological conditions in novel habitats above barriers to anadromy influence life‐history evolution. We highlight the importance of considering the adaptive landscape in conservation and restoration programs for species living in highly modified habitats, particularly with respect to key life‐history traits.

## 1 Introduction

Many animal populations are threatened by human impacts and accelerating global climate change (Urban, 2015), which affect biodiversity at multiple scales: individual, population, species, community, and ecosystem (Rands et al., 2010). Species can respond to environmental change by moving to the most suitable habitat (Harrisson, Pavlova, Telonis‐Scott, & Sunnucks, 2014), or through rapid changes in their phenotype (Darimont et al., 2009). Rapid adaptation to novel environmental conditions can in turn lead to directional change in the frequency of variants in specific genomic regions (Houde, Fraser, & Hutchings, 2010; Lamaze, Garant, & Bernatchez, 2013; Pearse, Miller, Abadía‐Cardoso, & Garza, 2014; Reed et al., 2015). In addition, increased phenotypic plasticity may allow organisms in variable environments to match their phenotype with the optimum trait value (Tufto, 2000) leading to an increase in mean population fitness (Ezard, Prizak, & Hoyle, 2014). Thus, both intra‐ and interpopulation diversity can be important in a variable environment and contribute to long‐term population viability (i.e., the “portfolio effect,” Schindler et al., 2010; Carlson & Satterthwaite, 2011).

Wild fish populations are impacted by climate change as well as local anthropogenic modification, including dams, water diversions, and other development in riverine habitats. In western North America, much of the landscape has been heavily affected by anthropogenic impacts over the past century (Nehlsen, Williams, & Lichatowich, 1991). Barriers to migration and habitat fragmentation have many consequences for migratory populations, including reduced population sizes leading to increased genetic drift (Clemento, Anderson, Boughton, Girman, & Garza, 2009), as well as local adaptation and rapid evolution resulting from divergent selection for different phenotypes within each new habitat (Crozier et al., 2008; Keeley, Parkinson, & Taylor, 2007). As a result, migratory fish impacted by habitat fragmentation are a primary target of conservation efforts (NMFS, 2012; Brown et al., 2013).

Numerous rivers and streams drain into San Francisco Bay, almost all of which are impacted by humans via dams, water diversions, and estuary and coastal wetland modifications (Goals Project, 2015). These watersheds shelter the salmonid species *Oncorhynchus mykiss* (Walbaum)*,* a widespread fish native to western North America that has been introduced all over the world for recreational fishing (Pascual et al., 2001) and aquaculture (Cowx, 2005–2013). Steelhead, the anadromous form of the species, typically spend ~1–2 years in freshwater before undergoing “smoltification,” a suite of morphological, physiological, and behavioral changes, and migrating to the sea to grow for 1–3 years before finally returning to freshwater one or more times to spawn. The resident form of *O. mykiss*, known as rainbow trout, lives exclusively in freshwater and does not migrate to the sea. These two life‐history forms can coexist in the same river, with an essentially continuous spectrum of life‐history variation represented in some populations (Hayes et al., 2012; McPhee et al., 2007; Shapovalov & Taft, 1954). Currently, steelhead populations along the West Coast are managed as distinct population segments (DPSs), which are delineated based on geographic, ecological, and genetic variation (Federal Register, 2006). The San Francisco Bay steelhead populations are included in the Central California Coast Steelhead DPS (Federal Register, 2006), which is distinct from the Central Valley Steelhead DPS, whose members migrate through San Francisco Bay during their anadromous migrations from the Sacramento and San Joaquin Rivers. Importantly, under the DPS system, only the migratory form of the species receives protection under the US Endangered Species Act (ESA; Federal Register, 2006), highlighting the critical importance of understanding the genetic basis of life‐history variation in this species (Pearse et al., 2014).

Migration is an important determinant of steelhead population structure, and numerous studies have examined the genetic structure of *O. mykiss* populations in California coastal and Central Valley watersheds (Arciniega et al., 2016; Clemento et al., 2009; Garza et al., 2014; Nielsen, Carpanzano, Fountain, & Gan, 1997; Nielsen, Pavey, Wiacek, & Williams, 2005; Pearse, Donohoe, & Garza, 2007; Pearse & Garza, 2015). Garza et al. (2014) highlighted the correlation between genetic variation and geographic distance, a relationship known as isolation by distance, which is influenced by major geographic features but has also changed over time (Pearse, Martinez, & Garza, 2011). When barriers to movement prevent migration among populations, genetic divergence increases. Similarly, artificial propagation and stocking of domesticated hatchery trout strains with non‐native ancestry can alter existing population genetic structure and also may negatively impact fitness in natural populations through introgression and loss of local adaptation (Champagnon, Elmberg, Guillemain, Gauthier‐Clerc, & Lebreton, 2012; McLean, Bentzen, & Quinn, 2003). Nonetheless, millions of juvenile hatchery trout are released each year in California (Leitritz, 1970). In general, these hatchery fish have reduced reproductive success and fitness relative to wild fish (McLean et al., 2003) and may contribute to the decline of wild populations (Augerot, 2005). Thus, the identification of native‐lineage populations isolated above dams is an important aspect of conservation genetic studies of these fish populations.

Expression of life‐history variation, such as the timing of migration and maturation, may differ in relation to the phylogeographic structure of populations (Arciniega et al., 2016; Vähä et al., 2011). This phenotypic variation may be influenced by a combination of plasticity and heritable variation in salmonid fishes, including *O. mykiss* (Abadía‐Cardoso, Anderson, Pearse, & Garza, 2013; Liedvogel, Åkesson, & Bensch, 2011; Phillis et al., 2016; Quinn, Kinnison, & Unwin, 2001). Such specific genomic regions have been linked to migratory timing in *Salmo salar* (e.g., *clock* gene, O'Malley, Ford, & Hard, 2010) as well as the differential expression of age at return in males and females (Barson et al., 2015). Similarly, rapid adaptation of specific genomic regions related to anadromy is expected in response to barriers to migration such as dams or waterfalls (Martínez, Garza, & Pearse, 2011). However, anadromous traits may persist above barriers, despite strong selection against this trait because of phenotypic plasticity, negative correlation with other traits (e.g., male maturation, Thrower, Hard, & Joyce, 2004), or because some aspects of the migratory life history are selectively favored despite the lack of access to the ocean. Martínez et al. (2011) identified a specific genomic region implicated in migratory behavior in *O. mykiss*. Subsequent analyses have characterized a large genomic region on chromosome Omy5, here referred to as the Omy5 Migration Associated Region (*MAR*), that is, strongly associated with life history of populations of steelhead and rainbow trout (Hecht, Thrower, Hale, Miller, & Nichols, 2012; Miller et al., 2012; Pearse et al., 2014). Adaptive genetic variation in this region is associated with both lineage‐specific and environmental differences among populations (Abadía‐Cardoso et al., 2016; Pearse & Garza, 2015). Interestingly, alleles associated with anadromous migration have been observed at relatively high frequencies in some populations isolated above dams (Pearse et al., 2014). Because dams create reservoirs behind them, they create a different selective environment than other above‐barrier habitats (i.e., waterfalls) and may support adfluvial populations in which fish migrate between stream and lake (reservoir) habitats (Holecek, Scarnecchia, & Miller, 2012). This potential for the evolution of migratory behavior in populations of *O. mykiss* above some dams has important implications for restoration and recovery efforts.

Here, we evaluate the population genetic structure of *O. mykiss* in the San Francisco Bay Area using a combination of 14 microsatellites and 92 single nucleotide polymorphism (SNP) loci and test for signals of local adaptation to migratory opportunities. First, we evaluate the evolutionary ancestry of San Francisco Bay Area populations to determine whether they are more closely related to the coastal steelhead or Central Valley evolutionary lineages, and whether the release of large numbers of hatchery rainbow trout of diverse origins in many of the reservoirs in the study area have affected the genetic structure of the species. Introductions and artificial propagation can have many consequences on native populations, and understanding them is important for establishing effective conservation and management strategies (McLean et al., 2003). Second, we use two SNP loci linked to the chromosome Omy5 *MAR* to test the hypothesis that genetic variation associated with migratory life history may be favoured above dams with large reservoirs that can support an adfluvial population (Holecek et al., 2012; Pearse et al., 2014). Together, these analyses will inform ongoing and future management of this protected species and provide insight into the potential for the evolutionary application of adaptive genomic variation in conservation.

## 2 Materials and Methods

### 2.1 Samples

**Figure 1:**
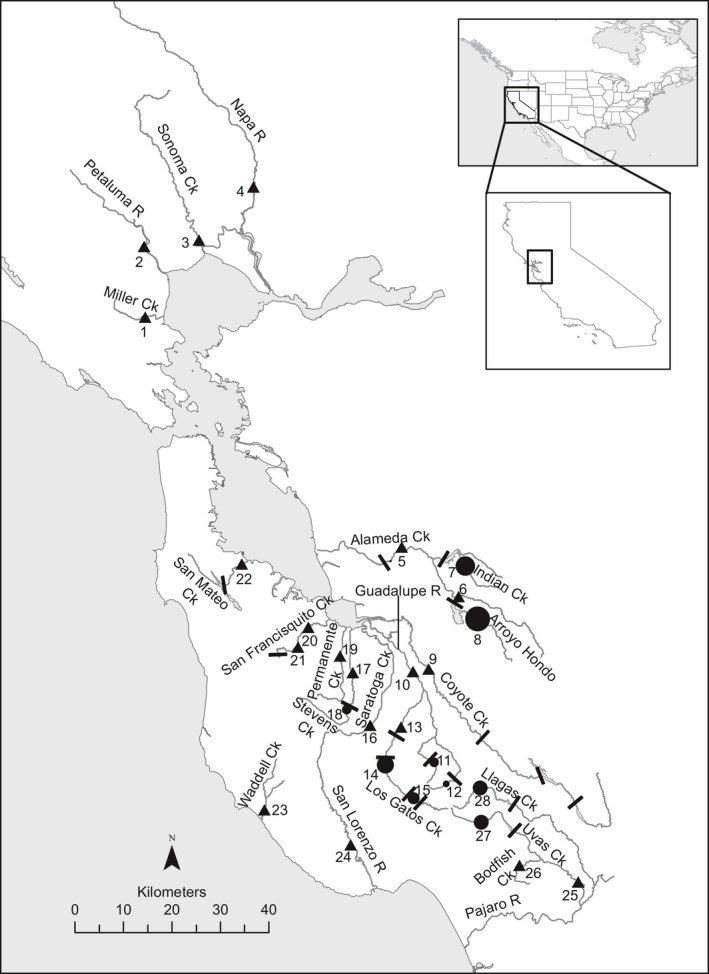
Map showing locations of population samples around San Francisco Bay. Sample sites are indicated by numbered triangles, and migration barriers are shown by bars, with sites above reservoir‐forming dams indicated by dots with size proportional to reservoir volume.

Adult and juvenile *O. mykiss* were sampled from 28 locations in six different areas around San Francisco Bay and from seven hatchery rainbow trout strains that have been stocked widely in California and elsewhere (Fig. 1; Table 1). Fish were captured from January 2002 to September 2013 using a variety of methods, including traps, electrofishing, and seine nets. Fish sampled in the same location in different years were combined for analyses. Tissue samples for DNA extraction consisted of a small piece of caudal fin (approximately 5 mm^2)^ desiccated on blotter paper until DNA extraction. In addition, previously published data (Abadía‐Cardoso et al., 2016; Clemento et al., 2009; Garza et al., 2014; Pearse & Garza, 2015) from populations of *O. mykiss* representing the Northern California (NC), California Central Valley (CCV), Central California Coast (CCC), and South‐Central California Coast (SCCC) DPSs were included in the analysis for comparison with San Francisco Bay Area populations (Table 2).

**Table 1:** Descriptive summary statistics for 28 populations of *Oncorhynchus mykiss* from basins in San Francisco (SF) Bay Area and the central California coast, as well as sampled hatchery rainbow trout strains

Region	#/Name	Barrier	N	H_E_	H_O_	N_A_	A_R_
North SF Bay	1/Miller Ck.^a^	Below	31	0.39	0.40	6.33	4.75
	2/Petaluma R.	Below	16	0.39	0.42	6.00	5.04
	3/Sonoma Ck.	Below	76	0.41	0.40	9.86	5.90
	4/Napa R.	Below	74	0.42	0.40	11.36	5.95
Alameda Creek	5/Alameda‐Stonybrook Ck.	Above	25	0.28	0.31	3.29	2.93
	6/Alameda‐Mainstem	Above	152	0.39	0.38	8.21	4.67
	7/Alameda‐Indian Ck.	Above	92	0.39	0.38	7.64	4.59
	8/Alameda‐Arroyo Hondo	Above	208	0.41	0.40	11.36	5.44
South SF Bay	9/Coyote Ck.	Below	129	0.40	0.38	12.00	5.82
	10/Guadalupe‐Mainstem	Below	141	0.40	0.39	9.07	4.78
	11/Guadalupe‐Reservoir	Above	20	0.39	0.41	5.43	4.54
	12/Guadalupe‐Herbert Ck.	Above	51	0.40	0.40	7.14	4.97
	13/Guadalupe‐Los Gatos Ck.	Below	62	0.41	0.42	8.57	5.54
	14/Guadalupe‐Lexington Res.	Above	30	0.39	0.35	6.79	4.78
	15/Guadalupe‐Austrian	Above	20	0.35	0.34	5.14	4.04
	16/Saratoga Ck.	Below	86	0.37	0.36	5.36	3.77
	17/Stevens Ck.	Below	17	0.40	0.40	6.71	5.59
	18/Stevens Res.	Above	20	0.37	0.36	6.86	5.34
	19/Permanente Ck.	Below	20	0.20	0.20	2.43	2.12
	20/San Francisquito Ck.^a^	Below	29	0.41	0.41	7.71	5.54
	21/San Francisquito‐Los Trancos Ck.	Below	24	0.39	0.40	6.38	4.69
West SF Bay	22/San Mateo Ck.	Below	96	0.41	0.40	8.36	4.78
Coastal/Monterey Bay	23/Waddell Ck.^a^	Below	31	0.40	0.40	7.36	4.85
	24/San Lorenzo R.^a^	Below	69	0.42	0.41	11.00	5.90
	25/Pajaro‐Uvas Ck.	Below	44	0.41	0.42	6.93	4.67
	26/Pajaro‐Bodfish Ck.	Below	57	0.42	0.44	8.14	5.21
	27/Pajaro‐Uvas Res.	Above	24	0.41	0.41	8.29	5.74
	28/Pajaro‐Chesbro Res.	Above	19	0.39	0.41	5.64	4.71
Hatchery Trout strains	Coleman^a^	–	47	0.37	0.36	6.43	4.42
	Virginia^a^	–	48	0.31	0.30	6.21	4.33
	Whitney^a^	–	48	0.36	0.36	6.43	4.14
	Wyoming^a^	–	47	0.37	0.38	6.29	4.51
	Kamloops^a^	–	47	0.27	0.27	7.29	4.50
	Eagle Lake^a^	–	47	0.29	0.28	5.00	3.84
	Mount Shasta^a^	–	47	0.35	0.34	5.07	3.60
			Mean	0.38	0.38	7.33	4.80

N, sample size; H_E_, expected heterozygosity; H_O_, observed heterozygosity; N_A_, observed number of alleles for microsatellite loci; A_R_, allelic richness for microsatellite loci. Barrier indicates the location of the sampling site as either A, above or B, below one or more impassable dams. Numbers (#) correspond to locations in Fig. 1. Ck., Creek; R., River; Res., Reservoir.

a The microsatellite and/or SNP data for these populations were previously published in Clemento et al. (2009), Garza et al. (2014), and/or Abadía‐Cardoso et al. (2016).

**Table 2:** *Oncorhynchus mykiss* populations from areas included in previous studies

Region	Tributary	DPS
North Coast	Mattole R.^a^	NC
	Gualala R.^a^	
Central Valley/Sacramento R.	North Fork American R.^b^	CCV
	Yuba R.^b^	
	McCloud R.^b^	
	Battle Ck.^b^	
	Deer Ck.^b^	
South‐Central Coast	Carmel R.^a^	SCCC
	Salinas R.‐Tassajara Ck.^c^	
	Big Sur R.^a^	
	Willow Ck.^a^	
Southern California	Santa Ynez R.‐Salsipuedes Ck.^c^	SC
	West Fork San Luis Rey^d^	

DPS indicates the distinct population segments.

NC, Northern California; CCV, California Central Valley; SCCC, South‐Central California Coast; SC, Southern California.

a Garza et al. (2014)

b Pearse and Garza (2015)

c Clemento et al. (2009)

d Abadía‐Cardoso et al. (2016)

### 2.2 Genetic data collection

Genomic DNA was extracted from all tissue samples using Qiagen DNeasy Tissue Kits on a BioRobot 3000 (Qiagen) following the manufacturer's protocols. Extracted DNA was diluted with dH_2_O at 20:1 for microsatellites and 2:1 for SNPs. Diluted samples were used for polymerase chain reaction (PCR) amplification of 15 microsatellite loci previously used in population genetic studies of *O. mykiss* (Garza et al., 2014). PCR was carried out in 15 μl volumes containing: 4 μl DNA template (1:20); 1.5 μl 10× Buffer, 0.95 μl MgCl_2_ (25 mM), 0.6 μl dNTPs (10 mM), 1 μl Primers (5 μM), 6.95 μl dH_2_O, and 0.045 μl Taq DNA polymerase. PCR products were mixed with formamide, loading dye, and an internal size standard, then denatured at 95°C for 3 min, and electrophoresed on an ABI 3730 DNA Analyzer (Applied Biosystems). All genotypes were called independently by two people using GeneMapper software (Applied Biosystems). Discrepancies were resolved by reconciling the two calls, by regenotyping, or by deletion of that genotype from the dataset.

All samples were also genotyped at 95 SNP loci previously developed and used for parentage and population genetic analysis in *O. mykiss* (Abadía‐Cardoso, Clemento, & Garza, 2011; Abadía‐Cardoso et al., 2013; Pearse & Garza, 2015), which include three SNPs located on chromosome Omy5, two of which are tightly linked, produced identical genotypes in almost all individuals, and are representative of the Omy5 *MAR* haplotypes (Pearse et al., 2014). Genotypes were obtained using TaqMan assays (Applied Biosystems) on 96.96 Dynamic SNP Genotyping Arrays in the EP1 Genotyping System (Fluidigm). Pre‐amplification PCR was performed with the diluted DNA at 2:1, and then, the pre‐amplification mix was diluted 4:1 with 2 μM Tris. The sample mix contained the following: 2.5 μl of the pre‐amp mix as template, 2.5 μl TaqMan Universal Master Mix, 2.5 μl 20 ×  Loading Reagent, 0.10 μl dH_2_O and 0.05 μl AmpliTaq Gold enzyme (Applied Biosystems). The assay mix contained 1.3 μl TaqMan Assay, 2.5 μl Assay Loading Reagent, 0.25 μl ROX and 1 μl dH_2_O. Assays and samples were loaded into the array, with two no‐template negative controls included in each genotyping array. Genotypes were called using Fluidigm SNP Genotyping Analysis Software v3.1.1.

### 2.3 Data analysis

The microsatellite and SNP data from San Francisco Bay *O. mykiss* were combined with data previously collected from a range of California populations, as well as hatchery rainbow trout strains commonly used in California to evaluate their influence on population structure. We first removed the two loci located in the Omy5 *MAR* (Omy11448‐87, Omy121006‐131), as well as a third locus (Omy127236‐583) that is partially linked to these loci (Pearse et al., 2014). The microsatellite locus Ots3M was also removed due to missing data, leaving a final set of 106 loci (92 SNP loci and 14 microsatellites) for further analysis. Observed heterozygosity (H_O_), expected heterozygosity (H_E_; Nei, 1987), and number of alleles were calculated for each population with the Microsatellite Toolkit (Park, 2001), and allelic richness (A_R_) was calculated to account for sample size effects using the rarefaction method in the program HP‐RARE and a base sample of 16 gene copies (Kalinowski, 2005). Pairwise F_ST_ values between all pairs of San Francisco Bay populations were calculated using the θ estimator of Weir and Cockerham (1984) in Genetix (Belkhir, Borsa, Chikhi, Raufaste, & Bonhomme, 1996), with 100 permutations to test whether values were significantly different from zero.

To identify ancestry and population structure and assign individual fish to their population of origin, we used two analysis methods based on individual genotypes rather than on population samples. First, clustering analyses were conducted using a Bayesian model‐based method implemented in the program *structure v2*.2 (Pritchard, Stephens, & Donnelly, 2000), in which each individual's genotype is fractionally assigned to a hypothesized number of genetic groups, *K*, without regard of the original geographic location. Analyses were conducted over a range of values from *K *=* *2–15. Multiple runs were performed for each *K* value to evaluate consistent patterns of genetic association, and the optimal number of genetic groups was evaluated using the method of Evanno, Regnaut, and Goudet (2005) implemented in STRUCTURE HARVESTER (Earl & vonHoldt, 2011). Results from *structure* consist of individual proportional assignments (Q values) to each *K* genetic cluster and were visualized using the programs *CLUMPP* and *distruct* (Jakobsson & Rosenberg, 2007; Rosenberg, 2004). To complement the *structure* analysis, an individual‐based principal component analysis (PCA) was performed with the R package “adegenet” (Jombart, 2008), combining the multivariate information into a few synthesized variables.

Unrooted neighbor‐joining trees were constructed using matrices of chord genetic distance (Cavalli‐Sforza & Edwards, 1967), using the software package *PHYLIP* v3.5c (Felsenstein, 1993). For this analysis, an additional two microsatellite and three SNP loci were removed because of missing data in one of the populations. Tree topology was determined by the neighbor‐joining algorithm, and the *consense* program of *PHYLIP* was used to perform 1000 bootstraps of the distance matrix. The resulting trees were visualized using *TreeView* (Page, 2001).

Finally, patterns of variation on chromosome Omy5 were evaluated based on the two loci known to be under selection and associated with life‐history patterns in coastal California populations (Omy114448‐87 and Omy121006‐131). These two loci are in near‐perfect linkage disequilibrium (LD) in coastal‐lineage *O. mykiss* (Abadía‐Cardoso et al., 2011; Pearse et al., 2014), but have reduced LD in at least some Central Valley lineage populations of *O. mykiss* (Abadía‐Cardoso et al., 2016; Pearse & Garza, 2015). Thus, to further evaluate the association between these two loci, the discordance in genotype and allele frequency estimates for all populations were compared and LD, which represents the nonrandom association of alleles at different loci (Slatkin, 2008), was estimated via the correlation coefficient (r^2^) between the two Omy5 markers for each population using the R package *genetics* (Warnes & Leisch, 2006). For these analyses, the Kamloops hatchery strain and McCloud River population sample were removed, as all individuals were monomorphic at Omy114448 and/or Omy121006, so LD between them could not be evaluated. Finally, to quantify the association between habitat type and adaptive genomic variation, the frequencies of alleles at the linked Omy5 loci were used as an indicator of Omy5 *MAR* resident (RS)‐ and anadromous (AD)‐associated haplotypes (Abadía‐Cardoso et al., 2016; Pearse & Garza, 2015; Pearse et al., 2014). For all of the above, comparisons between pair and group mean values of diversity statistics were performed with t‐tests (Student 1908) and ANOVA in the R software package (R Core Team, 2015).

## 3 Results

A total of 1,419 genotypes from San Francisco Bay Area populations were included in the present analysis (Table 1). These data were combined with genotype data from 331 fish representing hatchery rainbow trout strains and 704 fish from Central Valley, Monterey Bay, and other coastal California streams. Thus, a combined dataset of genotypes for 2,454 individuals was used in this study.

### 3.1 Population genetic diversity

Observed (H_O_) and expected (H_E_) heterozygosity ranged from 0.20–0.42 and 0.20–0.44, respectively (Table 1), with mean values of 0.38 and 0.38, respectively, over the 34 population samples evaluated. Allelic richness (A_R_) ranged from 2.12 (Permanente Creek) to 5.95 (Napa River). Mean H_O,_ H_E_, and A_R_ were not significantly different between populations above and below dams, but were all significantly higher in wild San Francisco Bay populations than in the hatchery trout strains (H_O_: 0.38 vs. 0.32, *p* < .05; H_E_: 0.38 vs. 0.33, *p* < .05; A_R_: 4.92 vs. 4.19, *p* < .05). Pairwise F_ST_ values among all populations ranged from 0.030–0.095, and all values were significantly different from zero. As expected, given the known relationship between genetic diversity and F_ST_ (Pearse et al., 2007), there was a strong negative correlation between population mean pairwise F_ST_ values and allelic richness (R^2^ = .90).

### 3.2 Individual analyses

**Figure 2:**
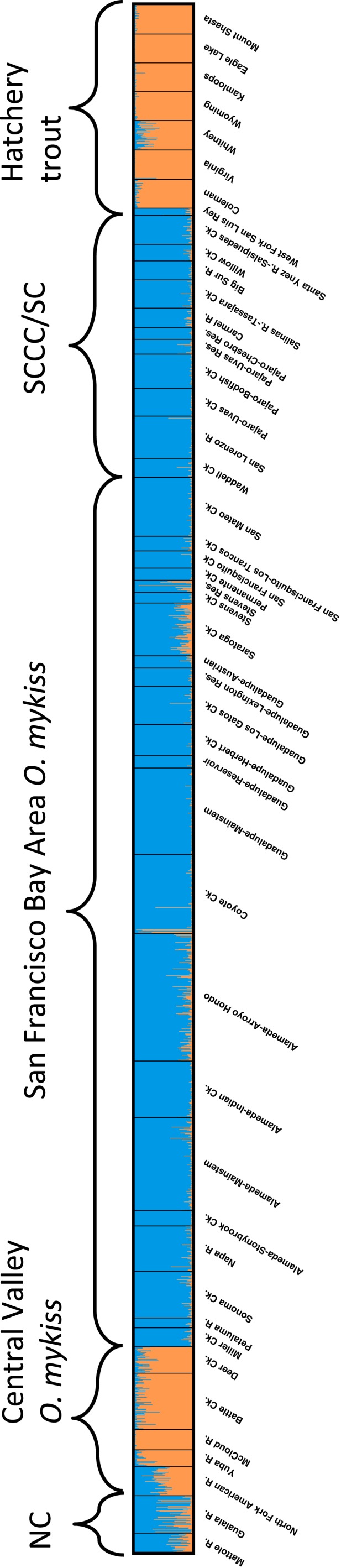
Plots from the program *distruct* representing *Structure* runs for neutral loci with K = 2. Each individual is represented by a single vertical line, with colors indicating their proportional ancestry in two genetic groups

**Figure 3:**
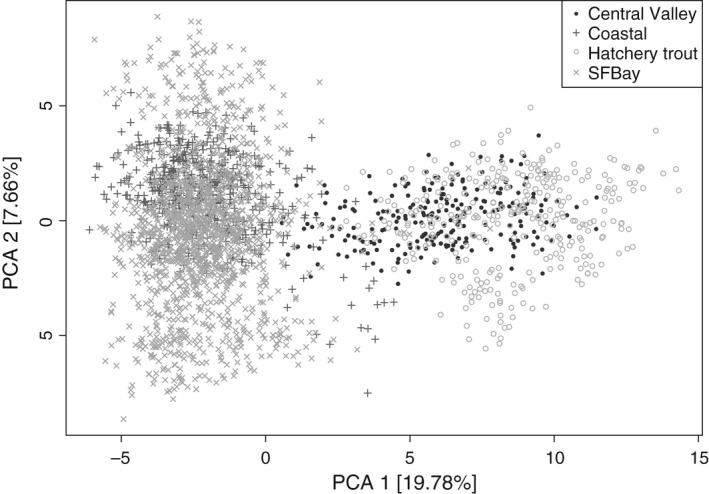
Principal component analysis showing relative genetic distances between individuals with coastal, San Francisco (SF) Bay Area, Central Valley, and hatchery trout strain ancestry

Individual‐based analysis with the program *structure* identified ancestry and assigned fish to their populations of origin. In general, consistent patterns of variation were observed across runs with *K *=* *2–15, and the number of cluster groups that best fit the data based on the Evanno method was *K *=* *2. For *K *=* *2, all 10 runs converged to the same result, with an abrupt shift in ancestry observed between coastal and Central Valley lineages (Fig. 2). All San Francisco Bay Area populations shared strong ancestry with the coastal lineage, while hatchery trout strains are clearly derived from the Central Valley lineage (Fig. 2). Six individuals in the Coyote Ck. population and one individual in Stevens Ck. reservoir had significant Central Valley lineage ancestry, and there were weak signals of Central Valley ancestry in some other populations, most notably Saratoga Ck. Results with higher values of *K* were also concordant with these overall patterns of population genetic associations. The principal component analysis (PCA) for the San Francisco Bay populations, hatchery strains, and coastal and Central Valley populations showed a similar, clear primary dichotomy between coastal/San Francisco Bay and hatchery/Central Valley fish (Fig. 3).

### 3.3 Population analyses

**Figure 4:**
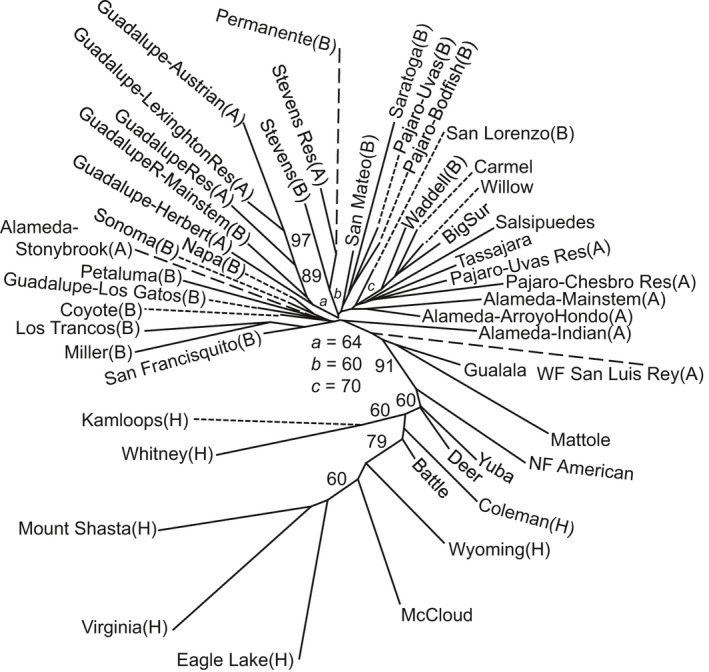
Unrooted neighbor‐joining tree constructed with chord distances from population samples described in Tables 1 and 2. Bootstrap values show percent out of 1,000 replicates, with only values >50% reported. For San Francisco Bay Area populations, A=above barrier and B=below barrier, and H=hatchery trout. Dashed lines are approximately half the actual branch length, and dotted lines connect population names to branch tips

Phylogeographic analysis with the full dataset revealed strong clustering separating coastal and San Francisco Bay populations from all hatchery strains and Central Valley populations (Fig. 4). All San Francisco Bay Area populations clustered closely together, indicating low genetic differentiation among them, and strong bootstrap support was observed among most populations within the same basin, including those above and below barrier dams (Fig. 4).

### 3.4 Chromosome Omy5

**Figure 5:**
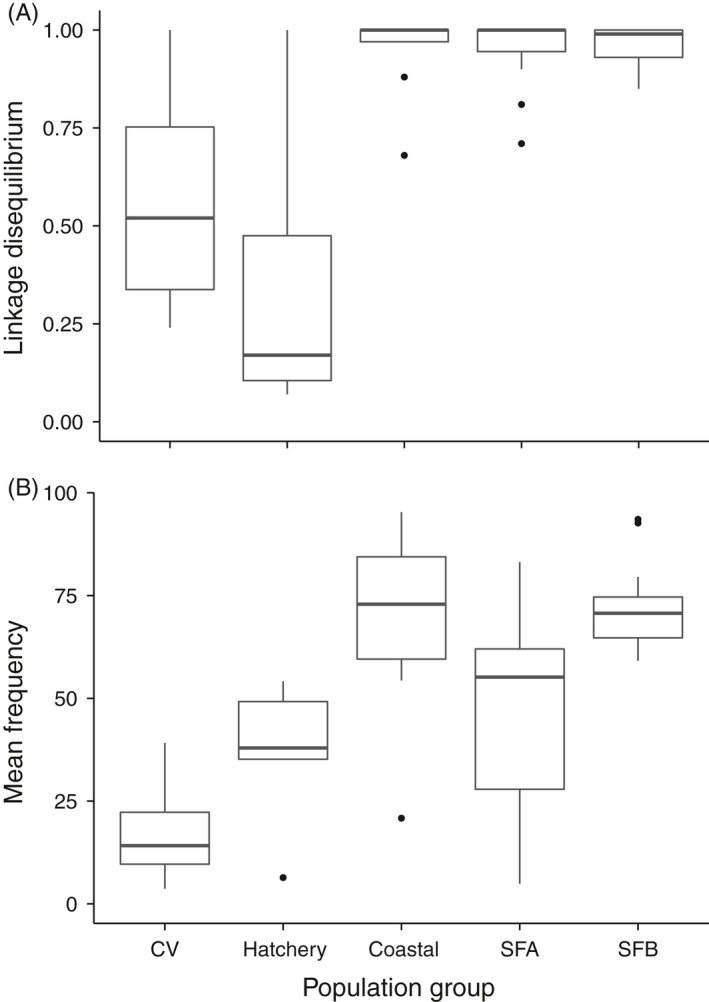
Mean values of (A) linkage disequilibrium (r^2^) and (B) frequency of alleles associated with migratory behavior on chromosome Omy5 for five groups of populations: Central Valley (CV), hatchery trout strains, coastal steelhead, and San Francisco Bay above‐ (SFA) and below‐barrier (SFB) populations

**Figure 6:**
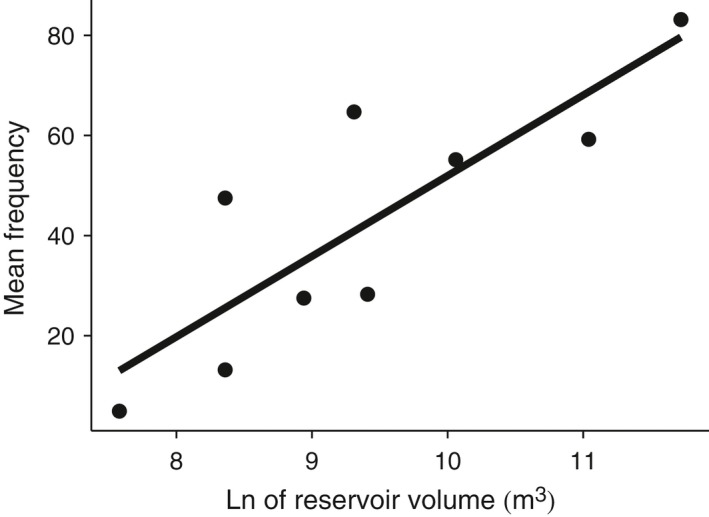
Relationship between the nominal capacity of surveyed reservoirs and the observed frequency of anadromy‐associated alleles for chromosome Omy5 loci (R^2^=.69, *p* < .01). Reservoir volume data were obtained from California Department of Water Resources (DWR 2015)

Linkage disequilibrium (r^2^) between the two loci located in the Omy5 *MAR* ranged from 1 (perfect linkage) to <0.1 (very low) and was significantly lower in Central Valley populations and hatchery strains than in coastal or San Francisco Bay Area populations, both above and below barriers (ANOVA, *p* ⪡ .001, Fig. 5a). Similarly, allele frequencies for the Omy5 *MAR* loci varied significantly among population groups (ANOVA, *p* ⪡ .001, Fig. 5b). Within the San Francisco Bay Area, most above‐barrier populations had low frequencies of alleles associated with anadromy, and thus the migratory AD haplotype, while significantly higher frequencies were consistently observed in below‐barrier populations (above = 49.50 and below = 71.31, *p* < .05, Fig. 5b). However, most barriers in the present study were formed by dams that created reservoirs rather than being natural waterfall barriers, and several of these above‐dam populations possessed relatively high frequencies of the anadromy‐associated Omy5 alleles. Among these, a strong correlation was observed between nominal reservoir volume and Omy5 migratory (AD) haplotype frequency among the nine above‐barrier populations that had access to a downstream reservoir (R^2^ = .69, *p* < .01; Fig. 6).

## 4 Discussion

Evaluation of microsatellite and SNP marker data demonstrated that San Francisco Bay Area *O. mykiss* populations are primarily derived from native coastal steelhead ancestry, and no significant hatchery rainbow trout introgression was detected in any population. This pattern is supported by (i) *structure* clustering and PCA analyses showing that fish from San Francisco Bay Area streams align with coastal populations; (ii) the unrooted neighbor‐joining tree, in which San Francisco Bay Area populations branch with coastal populations to the exclusion of Central Valley populations and hatchery rainbow trout strains; and (iii) the strong linkage disequilibrium between two loci located in the Omy5 Migration Associated Region (*MAR*), which is similar to the LD observed in coastal *O. mykiss* (Abadía‐Cardoso et al., 2016; Pearse & Garza, 2015; Pearse et al., 2014).

The population genetic results clearly distinguish all San Francisco Bay populations from the hatchery trout strains and Central Valley populations, which cluster together since these trout strains were domesticated primarily from Central Valley source populations (Busack & Gall, 1980). Thus, the original *O. mykiss* populations trapped upstream during dam construction have not been significantly introgressed or replaced by non‐native hatchery trout despite the extensive history of stocking above some dams in the study area. Moreover, San Francisco Bay Area *O. mykiss* populations above and below barriers within the same basin are generally more closely related to each other than to populations from other streams. These results are consistent with the general patterns observed in most coastal steelhead populations (Clemento et al., 2009; Pearse et al., 2009). In contrast, a recent study of Central Valley *O. mykiss* populations found a general lack of similarity between above and below barriers in the same basin and highly modified population structure (Pearse & Garza, 2015). Similarly, a recent study in Southern California found many cases in which *O. mykiss* populations with native ancestry had been completely replaced by hatchery rainbow trout (Abadía‐Cardoso et al., 2016). Thus, although the evolutionary impacts of hatchery trout stocking on native populations vary among locations, our study strongly confirms the native coastal ancestry of *O. mykiss* in the streams of the San Francisco Bay Area and also suggests that the decreased isolation by distance seen in modern coastal California *O. mykiss* relative to historical museum specimens (Pearse et al., 2011) results primarily from habitat fragmentation rather than introgression by hatchery trout.

In addition to the presumably neutral genetic markers used in the population genetic analysis, we assayed two SNP loci located in the *MAR* on chromosome Omy5, a large genomic region that is strongly associated with the expression of migratory life‐history traits in coastal steelhead (Martínez et al., 2011; Pearse et al., 2014). We found strong linkage disequilibrium (LD) between the two Omy5 *MAR* loci for all coastal and San Francisco Bay populations, but reduced LD in the Central Valley populations and hatchery trout, consistent with previous studies using these loci (Abadía‐Cardoso et al., 2016; Pearse & Garza, 2015) and supporting our conclusion based on neutral genetic markers that San Francisco Bay populations are of predominantly coastal origin. These results confirm and extend the finding that the *MAR* haplotypes represent a large block of linked loci on chromosome Omy5 (Pearse et al., 2014), likely maintained by a chromosomal inversion (Lowry & Willis, 2010).

Patterns of *Omy5 MAR* allele frequency variation among San Francisco Bay Area *O. mykiss* populations were similar to those seen in coastal steelhead (Abadía‐Cardoso et al., 2016; Pearse & Garza, 2015; Pearse et al., 2014), with a significantly lower frequency of the AD haplotype in above‐barrier populations compared with those below barriers to migration. However, several above‐barrier populations had a relatively high frequency of anadromy‐associated alleles. Natural waterfall barriers stop upstream fish migration, but fish may freely pass downstream over the falls, while fish remaining above the falls must adopt a resident life history (Phillis et al., 2016). Thus, a decrease of the anadromy‐associated AD haplotype, and conversely an increase of the RS haplotype, is expected in natural above‐barrier populations. In contrast, there are fundamental differences in the selective environment of populations above dams with reservoirs. First, fish may be physically unable to pass downstream over the dam and out of the reservoir, except during rare spill events, preventing them from leaving the population. In addition, fish may be able to exploit reservoirs as feeding and rearing habitat, particularly large reservoirs, migrating upriver to spawn as adfluvial trout (Holecek et al., 2012; Thrower, Joyce, Celewycz, & Malecha, 2008). Finally, ecological conditions in streams flowing into the reservoir may not support resident trout, reducing the relative reproductive success of fish that do not migrate down to the reservoir.

Our results support the hypothesis that access to a reservoir for *O. mykiss* populations above dams can lead to retention of the genetic variants and migratory behavior associated with anadromy. For example, trout in Arroyo Hondo and Indian Creek, which flow into the large Calaveras and San Antonio Reservoirs of Alameda Creek, respectively, are known to display adfluvial migration (Leidy, Becker, & Harvey, 2005; SFPUC 2009), and these populations had the highest observed frequencies of Omy5 MAR migratory alleles. Together, these results support the hypothesis that the presence of large reservoirs, along with other environmental factors, has the potential to support an adfluvial life history in *O. mykiss* populations trapped above dams, which may be particularly important for females (Rundio et al. 2012). It is interesting to note that, unlike the environment encountered by truly anadromous fish, adfluvial reservoir trout remain entirely in freshwater, so the physiological osmoregulatory switch to a saltwater environment must be decoupled from the rest of this complex migratory phenotype. Smoltification involves morphological, physiological, and behavior changes regulated by a complex genetic network, including the large *MAR* on chromosome *Omy5,* candidate loci located on chromosomes *Omy10*,* Omy12,* and *Omy14,* among others (e.g., Hecht et al., 2012; Martínez et al., 2011)*,* and also has the potential for epigenetic control (Baerwald et al., 2015) and differential gene expression (McKinney et al., 2015). It is important to note that the markers used here are not the causative genetic loci on *Omy5* and in fact may not be in perfect linkage with the causative loci, as their LD is reduced in the Central Valley populations (Pearse & Garza, 2015). Thus, the genomic mechanism for the physiological decoupling in these adfluvial populations remains unknown. A draft rainbow trout genome has recently been published (Berthelot et al., 2014), and additional work on an improved assembly is underway to further characterize this region of *Omy5*.

### 4.1 Conservation implications

San Francisco Bay Area watersheds and streams have been heavily impacted by human habitat alterations, but are also the subject of significant restoration efforts at both local and regional scales (Goals Project, 2015). The US Endangered Species Act (ESA) has protected steelhead since 1997, but only naturally spawned, anadromous individuals below natural and artificial migration barriers benefit from this protection. In this study, *O. mykiss* populations above and below barriers in the same San Francisco Bay Area watersheds showed shared ancestry and close genetic relationships with each other, indicating a lack of introgression from non‐native rainbow trout or hatchery steelhead. In addition to their native ancestry, some above‐barrier fish populations upstream of large reservoirs still possess adaptive genomic variation associated with anadromy, highlighting their importance for the conservation of life‐history variation in this species. To maintain the potential for populations to adapt to future changes, it is crucial to conserve both neutral and adaptive genetic diversity such as the adaptive genomic variation represented by the Omy5 *MAR*. Ultimately, efforts to conserve steelhead populations could potentially benefit by explicitly using information about such variation to inform conservation and recovery planning (Pearse, 2016).

## Data Archiving Statement

All data for this study are permanently archived at the Southwest Fisheries Science Center Fisheries Ecology Division ( https://swfsc.noaa.gov/FED/).
